# *N*-(4-Chloro­phen­yl)-3,4,5-trimethoxy­benzamide

**DOI:** 10.1107/S1600536808023234

**Published:** 2008-07-31

**Authors:** Aamer Saeed, Rasheed Ahmad Khera, Mahira Batool, Uzma Shaheen, Ulrich Flörke

**Affiliations:** a Department of Chemistry, Quaid-i-Azam University, Islamabad, Pakistan; b Department Chemie, Fakultät für Naturwissenschaften, Universität Paderborn, Warburgerstrasse 100, D-33098 Paderborn, Germany

## Abstract

In the title compound, C_16_H_16_ClNO_4_, the dihedral angle between the two aromatic rings is 67.33 (8)°. The crystal packing shows strong inter­molecular N—H⋯O hydrogen bonds that link the mol­ecules to form chains along [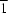
01].

## Related literature

For related literature, see: Capdeville *et al.* (2002 ▶); Ho *et al.* (2002 ▶); Igawa *et al.* (1999 ▶); Jackson *et al.* (1994 ▶); Makino *et al.* (2003 ▶); Zhichkin *et al.* (2007 ▶).
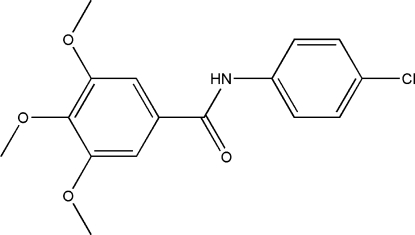


## Experimental

### 

#### Crystal data

C_16_H_16_ClNO_4_
*M* *_r_* = 321.75
Monoclinic, 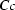

*a* = 9.487 (2) Å
*b* = 25.666 (6) Å
*c* = 6.9781 (15) Å
β = 112.340 (5)°
*V* = 1571.5 (6) Å^3^
*Z* = 4
Mo *K*α radiation
μ = 0.26 mm^−1^
*T* = 120 (2) K
0.41 × 0.10 × 0.10 mm

#### Data collection

Bruker SMART APEX diffractometer
Absorption correction: multi-scan (*SADABS*; Sheldrick, 2004 ▶) *T*_min_ = 0.901, *T*_max_ = 0.975
6765 measured reflections
3581 independent reflections
3104 reflections with *I* > 2σ(*I*)
*R*_int_ = 0.040

#### Refinement

*R*[*F* ^2^ > 2σ(*F* ^2^)] = 0.045
*wR*(*F* ^2^) = 0.106
*S* = 1.02
3581 reflections
202 parameters
2 restraints
H-atom parameters constrained
Δρ_max_ = 0.33 e Å^−3^
Δρ_min_ = −0.24 e Å^−3^
Absolute structure: Flack (1983 ▶), with 1780 Friedel pairs
Flack parameter: 0.06 (6)

### 

Data collection: *SMART* (Bruker, 2002 ▶); cell refinement: *SAINT* (Bruker, 2002 ▶); data reduction: *SAINT*; program(s) used to solve structure: *SHELXS97* (Sheldrick, 2008 ▶); program(s) used to refine structure: *SHELXL97* (Sheldrick, 2008 ▶); molecular graphics: *SHELXTL* (Sheldrick, 2008 ▶); software used to prepare material for publication: *SHELXTL*.

## Supplementary Material

Crystal structure: contains datablocks I, global. DOI: 10.1107/S1600536808023234/bt2747sup1.cif

Structure factors: contains datablocks I. DOI: 10.1107/S1600536808023234/bt2747Isup2.hkl

Additional supplementary materials:  crystallographic information; 3D view; checkCIF report

## Figures and Tables

**Table 1:** Hydrogen-bond geometry (Å, °)

*D*—H⋯*A*	*D*—H	H⋯*A*	*D*⋯*A*	*D*—H⋯*A*
N1—H1⋯O1^i^	0.88	2.18	2.878 (3)	136

Symmetry code: (i) 
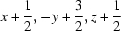
.

**Fig. 1.**
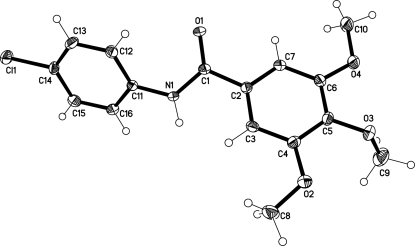
Molecular structure of title compound. Displacement ellipsoids are drawn at the 50% probability level.

**Fig. 2.**
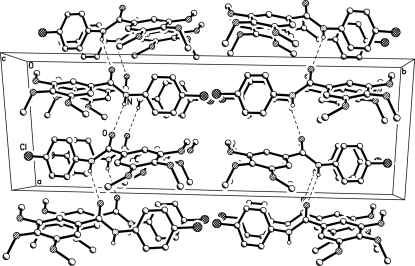
Crystal packing viewed along [001] with intermolecular hydrogen bonding pattern indicated as dashed lines. H-atoms not involved in hydrogen bonding are omitted.

## supplementary crystallographic information

### Comment

The benzanilide core is present in compounds with a wide range of biological activities that it has been called a privileged structure. Benzanilides serve as intermediates towards benzothiadiazin-4-ones (Makino *et al.*, 2003), benzodiazepine-2,5-diones (Ho *et al.*, 20022), and 2,3-disubstituted 3*H*-quinazoline-4-ones (Zhichkin *et al.*, 2007). Benzanilides have established their efficacy as centroid elements of ligands that bind to a wide variety of receptor types. Thus benzanilides containing aminoalkyl groups originally designed as a peptidomimetic, have been incorporated in an Arg-Gly-Asp cyclic peptide yielding a high affinity GPIIb/IIIa ligand (Jackson *et al.*, 1994). Imatinib is an ATP-site binding kinase inhibitor and platelet-derived growth factor receptor kinases (Capdeville *et al.*, 2002). Benzamides have activities as acetyl-CoA carboxylase and farnesyl transferase inhibitors (Igawa *et al.*, 1999)

Geometric parameters of the title compound, C_16_H_16_ClNO_4_, are in the usual ranges. The dihedral angle between the two aromatic rings is 67.33 (8)° and the torsion angles N1—C1—C2—C3 and C1—N1—C11—C12 are -31.1 (3)° and -39.2 (4)°, respectively. Of the three methoxy groups two of them lie nearly in plane with the aromatic ring, the O(3) group is almost perpendicular with C9—O3—C5—C4 of 92.2 (3)°. The crystal packing shows strong intermolecular N—H···O  bonds that link molecules to endless chains along [-101]. Details are given in Table 1.

### Experimental

Trimethoxybenzoyl chloride (5.4 mmol) in CHCl_3_ was treated with 4-chloroaniline (21.6 mmol) under a nitrogen atmosphere at reflux for 4 h. Upon cooling, the reaction mixture was diluted with CHCl_3_ and washed consecutively with aq 1 *M* HCl and saturated aq NaHCO_3_. The organic layer was dried over anhydrous sodium sulfate and concentrated under reduced pressure. Crystallization of the residue in CHCl_3_ afforded the title compound (84%) as white needles: IR (KBr) 3226, 1665, 1616, 1520, 1352 cm^-1^; 1H NMR (CDCl_3_, 400 MHz) δ 8.13 (d, J = 8 Hz, 1H), 7.81 (d, J = 8 Hz, 1H), 7.51 (dd, J = 8 Hz, 1H), 7.66 (dd, J = 8 Hz, 1H), 7.43 (d, J = 8 Hz, 2H), 7.36 (br s, 1H), 7.25 (d, J = 8 Hz, 1H), 3.89 (9H, s, OMex3). Anal. Calcd. For C_16_H_16_ClNO_4_, C, 59.73; H, 5.01; 11.02; N, 4.35; found C, 59.69; H, 5.04; 11.02; N, 4.42

### Refinement

Hydrogen atoms were located in difference syntheses, refined at idealized positions riding on the carbon or nitrogen atoms with isotropic displacement parameters *U*_iso_(H) = 1.2U_eq_(C or N) and 1.5U for methyl-C.

### Crystal data

**Table:**

C_16_H_16_ClNO_4_	*F*_000_ = 672
*M**_r_* = 321.75	*D*_x_ = 1.360 Mg m^−^^3^
Monoclinic, *C**c*	Mo *K*α radiation λ = 0.71073 Å
Hall symbol: C -2yc	Cell parameters from 879 reflections
*a* = 9.487 (2) Å	θ = 2.5–26.6º
*b* = 25.666 (6) Å	µ = 0.26 mm^−^^1^
*c* = 6.9781 (15) Å	*T* = 120 (2) K
β = 112.340 (5)º	Prism, colourless
*V* = 1571.5 (6) Å^3^	0.41 × 0.10 × 0.10 mm
*Z* = 4	

### Data collection

**Table:**

Bruker SMART APEX diffractometer	3581 independent reflections
Radiation source: sealed tube	3104 reflections with *I* > 2σ(*I*)
Monochromator: graphite	*R*_int_ = 0.040
*T* = 120(2) K	θ_max_ = 27.9º
φ and ω scans	θ_min_ = 1.6º
Absorption correction: multi-scan(SADABS; Sheldrick, 2004)	*h* = −12→12
*T*_min_ = 0.901, *T*_max_ = 0.975	*k* = −33→29
6765 measured reflections	*l* = −9→9

### Refinement

**Table:**

Refinement on *F*^2^	Hydrogen site location: difference Fourier map
Least-squares matrix: full	H-atom parameters constrained
*R*[*F*^2^ > 2σ(*F*^2^)] = 0.045	*w* = 1/[σ^2^(*F*_o_^2^) + (0.0463*P*)^2^ + 0.1374*P*] where *P* = (*F*_o_^2^ + 2*F*_c_^2^)/3
*wR*(*F*^2^) = 0.106	(Δ/σ)_max_ < 0.001
*S* = 1.02	Δρ_max_ = 0.33 e Å^−^^3^
3581 reflections	Δρ_min_ = −0.24 e Å^−^^3^
202 parameters	Extinction correction: none
2 restraints	Absolute structure: Flack (1983), with 1780 Friedel pairs
Primary atom site location: structure-invariant direct methods	Flack parameter: 0.06 (6)
Secondary atom site location: difference Fourier map	

### Special details

**Table:**

Geometry. All e.s.d.'s (except the e.s.d. in the dihedral angle between two l.s. planes) are estimated using the full covariance matrix. The cell e.s.d.'s are taken into account individually in the estimation of e.s.d.'s in distances, angles and torsion angles; correlations between e.s.d.'s in cell parameters are only used when they are defined by crystal symmetry. An approximate (isotropic) treatment of cell e.s.d.'s is used for estimating e.s.d.'s involving l.s. planes.
Refinement. Refinement of *F*^2^ against ALL reflections. The weighted *R*-factor *wR* and goodness of fit *S* are based on *F*^2^, conventional *R*-factors *R* are based on *F*, with *F* set to zero for negative *F*^2^. The threshold expression of *F*^2^ > σ(*F*^2^) is used only for calculating *R*-factors(gt) *etc*. and is not relevant to the choice of reflections for refinement. *R*-factors based on *F*^2^ are statistically about twice as large as those based on *F*, and *R*- factors based on ALL data will be even larger.

### Fractional atomic coordinates and isotropic or equivalent isotropic displacement parameters (Å^2^)

**Table:**

	*x*	*y*	*z*	*U*_iso_*/*U*_eq_	
Cl1	0.24506 (8)	0.52096 (2)	−0.45259 (9)	0.03098 (17)	
O1	0.0700 (2)	0.75731 (7)	−0.2052 (3)	0.0251 (4)	
O2	0.3495 (2)	0.83040 (7)	0.6718 (3)	0.0293 (4)	
O3	0.2739 (2)	0.92522 (7)	0.5093 (3)	0.0287 (4)	
O4	0.1760 (2)	0.94204 (7)	0.1029 (3)	0.0293 (4)	
N1	0.2656 (2)	0.71265 (7)	0.0305 (3)	0.0206 (4)	
H1	0.3366	0.7136	0.1562	0.025*	
C1	0.1742 (3)	0.75494 (10)	−0.0350 (4)	0.0201 (5)	
C2	0.2087 (3)	0.79915 (9)	0.1153 (4)	0.0190 (5)	
C3	0.2689 (3)	0.79115 (10)	0.3293 (4)	0.0207 (5)	
H3A	0.2926	0.7570	0.3846	0.025*	
C4	0.2938 (3)	0.83381 (10)	0.4601 (4)	0.0217 (5)	
C5	0.2589 (3)	0.88383 (10)	0.3797 (4)	0.0217 (5)	
C6	0.2015 (3)	0.89163 (10)	0.1650 (4)	0.0226 (6)	
C7	0.1739 (3)	0.84890 (9)	0.0330 (4)	0.0209 (5)	
H7A	0.1314	0.8538	−0.1129	0.025*	
C8	0.4275 (4)	0.78426 (12)	0.7598 (5)	0.0380 (7)	
H8A	0.3560	0.7549	0.7223	0.057*	
H8B	0.4727	0.7878	0.9109	0.057*	
H8C	0.5083	0.7779	0.7073	0.057*	
C9	0.4195 (4)	0.94971 (13)	0.5730 (6)	0.0457 (8)	
H9A	0.4986	0.9251	0.6541	0.069*	
H9B	0.4213	0.9803	0.6582	0.069*	
H9C	0.4387	0.9606	0.4506	0.069*	
C10	0.1094 (4)	0.95144 (11)	−0.1151 (5)	0.0375 (7)	
H10A	0.1764	0.9377	−0.1807	0.056*	
H10B	0.0960	0.9890	−0.1405	0.056*	
H10C	0.0100	0.9341	−0.1736	0.056*	
C11	0.2554 (3)	0.66682 (9)	−0.0888 (4)	0.0194 (5)	
C12	0.1169 (3)	0.64558 (10)	−0.2157 (4)	0.0238 (5)	
H12A	0.0244	0.6618	−0.2258	0.029*	
C13	0.1133 (3)	0.60093 (10)	−0.3273 (4)	0.0269 (6)	
H13A	0.0184	0.5868	−0.4158	0.032*	
C14	0.2479 (3)	0.57674 (10)	−0.3103 (4)	0.0218 (5)	
C15	0.3862 (3)	0.59685 (10)	−0.1809 (4)	0.0259 (6)	
H15A	0.4784	0.5798	−0.1675	0.031*	
C16	0.3897 (3)	0.64200 (10)	−0.0706 (4)	0.0250 (6)	
H16A	0.4847	0.6561	0.0182	0.030*	

### Atomic displacement parameters (Å^2^)

**Table:**

	*U*^11^	*U*^22^	*U*^33^	*U*^12^	*U*^13^	*U*^23^
Cl1	0.0333 (3)	0.0268 (3)	0.0286 (3)	0.0033 (3)	0.0071 (3)	−0.0101 (3)
O1	0.0181 (9)	0.0240 (10)	0.0225 (9)	0.0011 (7)	−0.0043 (7)	−0.0031 (7)
O2	0.0373 (11)	0.0277 (10)	0.0193 (9)	0.0051 (8)	0.0066 (8)	0.0008 (8)
O3	0.0346 (11)	0.0236 (9)	0.0262 (10)	−0.0009 (8)	0.0097 (8)	−0.0075 (8)
O4	0.0406 (12)	0.0171 (9)	0.0248 (10)	0.0013 (8)	0.0062 (9)	0.0014 (8)
N1	0.0170 (10)	0.0186 (10)	0.0186 (10)	0.0016 (8)	−0.0019 (8)	−0.0021 (9)
C1	0.0157 (11)	0.0203 (12)	0.0207 (13)	−0.0004 (9)	0.0026 (11)	−0.0003 (10)
C2	0.0154 (12)	0.0179 (12)	0.0214 (13)	−0.0006 (9)	0.0046 (10)	−0.0017 (10)
C3	0.0172 (11)	0.0188 (12)	0.0221 (13)	−0.0002 (9)	0.0027 (10)	0.0033 (10)
C4	0.0218 (13)	0.0241 (14)	0.0184 (13)	−0.0003 (10)	0.0068 (11)	−0.0015 (10)
C5	0.0218 (12)	0.0203 (13)	0.0230 (13)	−0.0009 (10)	0.0085 (11)	−0.0056 (10)
C6	0.0227 (13)	0.0181 (13)	0.0248 (14)	0.0016 (10)	0.0065 (11)	0.0010 (10)
C7	0.0192 (11)	0.0211 (13)	0.0187 (12)	0.0010 (9)	0.0029 (10)	0.0005 (10)
C8	0.052 (2)	0.0397 (17)	0.0211 (14)	0.0166 (15)	0.0126 (14)	0.0076 (13)
C9	0.0401 (18)	0.0386 (18)	0.050 (2)	−0.0104 (14)	0.0079 (16)	−0.0163 (15)
C10	0.058 (2)	0.0190 (14)	0.0282 (15)	0.0046 (13)	0.0084 (14)	0.0044 (12)
C11	0.0225 (12)	0.0159 (12)	0.0161 (11)	−0.0007 (9)	0.0031 (10)	0.0018 (9)
C12	0.0176 (12)	0.0218 (13)	0.0294 (14)	0.0030 (10)	0.0059 (11)	−0.0023 (11)
C13	0.0200 (13)	0.0283 (15)	0.0255 (14)	−0.0029 (10)	0.0011 (11)	−0.0080 (11)
C14	0.0286 (13)	0.0161 (12)	0.0209 (13)	0.0003 (10)	0.0095 (11)	−0.0039 (10)
C15	0.0208 (13)	0.0263 (14)	0.0285 (14)	0.0057 (10)	0.0069 (11)	0.0015 (11)
C16	0.0191 (13)	0.0233 (14)	0.0255 (14)	−0.0013 (10)	0.0005 (11)	−0.0014 (11)

### Geometric parameters (Å, °)

**Table:**

Cl1—C14	1.737 (2)	C8—H8A	0.9800
O1—C1	1.225 (3)	C8—H8B	0.9800
O2—C4	1.369 (3)	C8—H8C	0.9800
O2—C8	1.408 (3)	C9—H9A	0.9800
O3—C5	1.367 (3)	C9—H9B	0.9800
O3—C9	1.426 (4)	C9—H9C	0.9800
O4—C6	1.357 (3)	C10—H10A	0.9800
O4—C10	1.429 (4)	C10—H10B	0.9800
N1—C1	1.356 (3)	C10—H10C	0.9800
N1—C11	1.423 (3)	C11—C16	1.386 (4)
N1—H1	0.8800	C11—C12	1.386 (4)
C1—C2	1.494 (3)	C12—C13	1.378 (4)
C2—C7	1.387 (3)	C12—H12A	0.9500
C2—C3	1.397 (4)	C13—C14	1.384 (4)
C3—C4	1.387 (3)	C13—H13A	0.9500
C3—H3A	0.9500	C14—C15	1.380 (4)
C4—C5	1.390 (4)	C15—C16	1.385 (4)
C5—C6	1.400 (3)	C15—H15A	0.9500
C6—C7	1.392 (4)	C16—H16A	0.9500
C7—H7A	0.9500		
			
C4—O2—C8	116.6 (2)	H8B—C8—H8C	109.5
C5—O3—C9	113.2 (2)	O3—C9—H9A	109.5
C6—O4—C10	117.0 (2)	O3—C9—H9B	109.5
C1—N1—C11	124.9 (2)	H9A—C9—H9B	109.5
C1—N1—H1	117.5	O3—C9—H9C	109.5
C11—N1—H1	117.5	H9A—C9—H9C	109.5
O1—C1—N1	123.0 (2)	H9B—C9—H9C	109.5
O1—C1—C2	121.6 (2)	O4—C10—H10A	109.5
N1—C1—C2	115.5 (2)	O4—C10—H10B	109.5
C7—C2—C3	120.9 (2)	H10A—C10—H10B	109.5
C7—C2—C1	117.0 (2)	O4—C10—H10C	109.5
C3—C2—C1	122.0 (2)	H10A—C10—H10C	109.5
C4—C3—C2	119.1 (2)	H10B—C10—H10C	109.5
C4—C3—H3A	120.5	C16—C11—C12	119.5 (2)
C2—C3—H3A	120.5	C16—C11—N1	118.1 (2)
O2—C4—C3	124.0 (2)	C12—C11—N1	122.4 (2)
O2—C4—C5	115.4 (2)	C13—C12—C11	120.1 (2)
C3—C4—C5	120.6 (2)	C13—C12—H12A	119.9
O3—C5—C4	120.1 (2)	C11—C12—H12A	119.9
O3—C5—C6	119.8 (2)	C12—C13—C14	120.0 (2)
C4—C5—C6	120.0 (2)	C12—C13—H13A	120.0
O4—C6—C7	125.0 (2)	C14—C13—H13A	120.0
O4—C6—C5	115.3 (2)	C15—C14—C13	120.3 (2)
C7—C6—C5	119.6 (2)	C15—C14—Cl1	119.1 (2)
C2—C7—C6	119.7 (2)	C13—C14—Cl1	120.5 (2)
C2—C7—H7A	120.1	C14—C15—C16	119.5 (2)
C6—C7—H7A	120.1	C14—C15—H15A	120.2
O2—C8—H8A	109.5	C16—C15—H15A	120.2
O2—C8—H8B	109.5	C15—C16—C11	120.5 (2)
H8A—C8—H8B	109.5	C15—C16—H16A	119.8
O2—C8—H8C	109.5	C11—C16—H16A	119.8
H8A—C8—H8C	109.5		
			
C11—N1—C1—O1	1.5 (4)	O3—C5—C6—O4	5.1 (3)
C11—N1—C1—C2	−178.5 (2)	C4—C5—C6—O4	−177.9 (2)
O1—C1—C2—C7	−29.2 (3)	O3—C5—C6—C7	−174.4 (2)
N1—C1—C2—C7	150.8 (2)	C4—C5—C6—C7	2.6 (4)
O1—C1—C2—C3	148.9 (2)	C3—C2—C7—C6	1.1 (4)
N1—C1—C2—C3	−31.1 (3)	C1—C2—C7—C6	179.2 (2)
C7—C2—C3—C4	0.0 (4)	O4—C6—C7—C2	178.2 (2)
C1—C2—C3—C4	−178.0 (2)	C5—C6—C7—C2	−2.4 (4)
C8—O2—C4—C3	19.9 (4)	C1—N1—C11—C16	143.0 (3)
C8—O2—C4—C5	−161.6 (3)	C1—N1—C11—C12	−39.2 (4)
C2—C3—C4—O2	178.7 (2)	C16—C11—C12—C13	−1.8 (4)
C2—C3—C4—C5	0.2 (4)	N1—C11—C12—C13	−179.6 (2)
C9—O3—C5—C4	92.2 (3)	C11—C12—C13—C14	1.0 (4)
C9—O3—C5—C6	−90.8 (3)	C12—C13—C14—C15	0.5 (4)
O2—C4—C5—O3	−3.1 (3)	C12—C13—C14—Cl1	−179.1 (2)
C3—C4—C5—O3	175.5 (2)	C13—C14—C15—C16	−1.1 (4)
O2—C4—C5—C6	179.8 (2)	Cl1—C14—C15—C16	178.5 (2)
C3—C4—C5—C6	−1.6 (4)	C14—C15—C16—C11	0.3 (4)
C10—O4—C6—C7	2.9 (4)	C12—C11—C16—C15	1.2 (4)
C10—O4—C6—C5	−176.6 (3)	N1—C11—C16—C15	179.1 (2)

### Hydrogen-bond geometry (Å, °)

**Table:**

*D*—H···*A*	*D*—H	H···*A*	*D*···*A*	*D*—H···*A*
N1—H1···O1^i^	0.88	2.18	2.878 (3)	136

Symmetry codes: (i) *x*+1/2, −*y*+3/2, *z*+1/2.
